# Pharmacogenetics and the Response to Antidepressants in Major Depressive Disorder

**DOI:** 10.3390/ph18091360

**Published:** 2025-09-11

**Authors:** Amanda Gollo Bertollo, Ricieri Mocelin, Zuleide Maria Ignácio

**Affiliations:** 1 Graduate Program in Neurosciences, Federal University of Santa Catarina, Florianópolis 88040-900, SC, Brazil; amandagollo@gmail.com; 2 Laboratory of Physiology, Pharmacology, and Psychopathology, Graduate Program in Biomedical Sciences, Federal University of Fronteira Sul, Chapecó 89815-899, SC, Brazil; 3 Translational Neuropsychobiology Laboratory, Graduate Program in Biomedical Sciences, Federal University of Fronteira Sul, Passo Fundo 99010-020, RS, Brazil; ricieri.mocelin@uffs.edu.br

**Keywords:** pharmacogenetics, major depressive disorder, antidepressants, genetic variations, personalized medicine

## Abstract

**Purpose:** Genetic polymorphisms within specific genes play a role in both the genetic predisposition to Major Depressive Disorder (MDD) and the variation observed in responses to antidepressant treatments. Pharmacogenetics examines how these polymorphisms affect medication response. This review highlights significant disparities in the pharmacogenetic influences on antidepressant response, with a focus on ethnic and sex-based differences. **Methods:** This review synthesizes findings from a comprehensive literature search conducted between 2000 and 2025. It utilized databases such as PubMed, Scopus, and Web of Science, using search terms including “pharmacogenetics”, “antidepressants”, “Major Depressive Disorder”, “CYP450”, “neuroplasticity”, and “genetic variations”. This review integrates pharmacogenetics with neurotransmitters and their transporters, neuroplasticity, growth factors, and the cytochrome P450 family, providing promising insights for personalized MDD treatment strategies. We analyzed and synthesized findings from over 50 relevant studies, focusing on those with a clear emphasis on genetic associations with antidepressant efficacy and adverse effects. **Results:** Pharmacogenetic analysis facilitates personalized antidepressant prescriptions by identifying key genetic variants that influence treatment outcomes. Specifically, variations in CYP2D6 and CYP2C19 can significantly impact drug metabolism and tolerability. A high percentage of patients with non-normal metabolizer phenotypes are predisposed to adverse drug reactions or ineffective responses. Furthermore, this review identifies significant ethnic and sex-based disparities in treatment response. For example, the L allele of the 5-HTTLPR polymorphism confers a higher likelihood of response and remission following SSRI treatment in white people compared to Asians. Additionally, in women, specific 5-HTTLPR polymorphisms have a more pronounced influence on mood and MDD pathophysiology, with a significant reduction in mood in response to tryptophan depletion. **Conclusions:** Integrating pharmacogenetic insights, encompassing genetic factors, neurotransmitter pathways, neuroplasticity, and the influence of ethnicity and sex, is crucial for developing personalized antidepressant treatment strategies. This will ultimately optimize patient recovery and minimize adverse effects.

## 1. Introduction

Pharmacogenetics, related to antidepressants, is an area of research focusing on how individual genetic variations influence the response to antidepressant medications. Given the significant variability in antidepressant efficacy among patients, personalizing treatment based on genetic profiles offers a more effective strategy for managing Major Depressive Disorder (MDD) [1].

The most commonly used classes of classic antidepressants are selective serotonin reuptake inhibitors (SSRIs), such as fluoxetine, sertraline, and citalopram; serotonin and norepinephrine reuptake inhibitors (SNRIs), such as duloxetine, desipramine, and maprotiline; and tricyclic antidepressants (TCAs), such as amitriptyline, imipramine, and clomipramine [2,3].

Despite available treatments, MDD exhibits a high rate of non-response or inadequate response to antidepressants, with less than 50% of the patients achieving adequate response to the initial classic antidepressant. Around 30% of the patients do not achieve remission of MDD symptoms despite multiple therapeutic attempts and are therefore classified as having treatment-resistant depression (TRD) [4], which can result in morbidities for patients, besides significant costs [5]. TRD is strongly associated with different factors, such as childhood adversity, traumatic events, and bullying victimization. However, it can affect individuals without these histories [6] and genetic factors that seem responsible for approximately 50% of the variance in response to antidepressants [7].

Pharmacogenetics offers an opportunity to identify in advance which antidepressants may be most effective for a patient based on their unique genetics, helping to avoid unsuccessful treatment attempts [5]. Two widely used methodologies for research in the area are hypothesis-based candidate analysis and hypothesis-free Genome-Wide Association Studies (GWASs). While candidate analysis is a more targeted approach focused on specific genes based on previous hypotheses, GWASs explore the genome in an undirected way, seeking to identify genetic associations on a large scale without needing prior knowledge about the genes involved. Additionally, pharmacogenetic studies enable the identification of potential heterogeneities and the creation of clinical genetic panels that correlate antidepressants with specific genes and variants [8,9,10].

Pharmacogenetic-guided antidepressant treatment personalization enhances medication efficacy and accelerates the identification of optimal therapeutic strategies, which is particularly crucial for patients with severe TRD who have not responded to conventional approaches. Consequently, pharmacogenetics facilitates more informed and targeted MDD treatment decisions, improving patient outcomes [11].

While foundational reviews by Porcelli et al. [12] and Keers and Aitchison [13] explored the pharmacogenetics of antidepressant response, they were published at a time when research was still grappling with inconsistent findings and modest effect sizes. The Porcelli review, for example, highlighted a focus on pharmacodynamic genes despite inconsistencies. In contrast, the Keers review noted the disappointing failure in replicating some findings and a strong need for standardized methodologies. Over the past decade, significant advances in both research and technology have generated a wealth of new data. This review distinguishes itself by providing a holistic and integrated perspective, synthesizing the fragmented literature on the complex interactions between genetic variations, neurobiological factors (such as neurotransmitter systems, neuroplasticity, and inflammation), and key demographic variables (including ethnicity and sex). By doing so, this review addresses a critical gap and offers a unique framework for understanding personalized MDD treatment.

## 2. Methodology

This narrative review synthesizes and critically evaluates the existing literature on the pharmacogenetics of antidepressant response in MDD, focusing on the intricate interplay between genetic variations, key neurobiological systems, and demographic factors such as ethnicity and sex. A comprehensive search was conducted on PubMed, Scopus, and Web of Science for studies published between 2000 and 2025. The search strategy involved a combination of keywords, including “pharmacogenetics”, “antidepressant”, “Major Depressive Disorder”, “genetic polymorphism”, “*5-HTTLPR*”, “*BDNF*”, “*CYP2D6*”, “*CYP2C19*”, “ethnicity”, and “sex”. Only peer-reviewed articles in English involving human subjects, including original research, meta-analyses, and comprehensive reviews that investigated the association between genetic polymorphisms and antidepressant efficacy or adverse effects, were included. Articles not directly related to the topic were excluded. The selected studies were then analyzed, and key data on study design, population demographics, genes, and findings were extracted and compiled.

## 3. Genetic Influence on Major Depressive Disorder and Antidepressant Treatment

The etiology of MDD is a complex interaction of genetic, biological, psychological, and environmental factors. Family history of depression, chemical imbalances in the brain, personality traits, history of traumatic events, chronic stress, lack of social support, limited access to resources, and low self-esteem are psychological factors that may contribute to the etiology of depression. These factors can also interact with genetic predisposition [14].

A genetic influence on MDD has been a subject of significant research, and several studies have explored the role of genetics in the development and treatment of this complex mental health condition. A GWAS involving 807,553 individuals, 246,363 with depression and 561,190 controls, identified 102 independent genetic variants and implicated 269 genes, supporting the polygenic nature of depression. Additionally, the study identified 15 gene sets associated with depression, encompassing individual genes and genetic pathways related to synaptic structure and neurotransmission, primarily within the prefrontal cortex [15].

Diseases with an inflammatory pathophysiology, such as depression, are more likely to develop due to genetic problems that control lipid metabolism and consequently affect inflammatory mechanisms. The literature indicates that people with MDD have altered levels of phospholipids, including arachidonic acid. At least one study has identified a shared genetic etiology between this biological alteration and MDD [16].

Polymorphisms of the mineralocorticoid receptor (*MR*) and glucocorticoid receptor (*GR*) genes also influence the risk of MDD, considering their influence on the levels of the biomarkers aldosterone and cortisol, both of which are altered in the disorder. The polymorphism of the *MR* and *GR* genes is associated with stress at the beginning of life, a risk situation for the development of MDD [17] because it stimulates changes that can even influence the response to antidepressants [18].

Polymorphisms of the ATP-binding cassette (ABC) *B1* gene, which encodes P-glycoprotein (P-gp), an ATP-driven efflux pump at the blood–brain barrier, were also associated with the physiopathology and the response to antidepressants [19]. It is essential to consider that carriers of the minor allele for rs2032583 with elevated plasma levels of antidepressant have more sleep-related side effects compared to homozygotes for the major allele with high plasma levels. It is suggested that the elevation in plasma levels may be related to a reduced or altered P-gp efflux activity in patients who are carriers of the minor allele, emphasizing that the plasma levels of antidepressants of the SSRI, SSNRi, or TCA classes should not exceed the recommended range to obtain an optimal therapeutic result [20].

## 4. Major Depressive Disorder and Pharmacogenetics Related to Neurotransmitters, Their Receptors, and Neurotransmitter Transporters

The monoaminergic hypothesis of depression suggests that a deficiency in certain monoaminergic neurotransmitters, particularly serotonin, norepinephrine, and dopamine, plays a fundamental role in the development and maintenance of depressive symptoms. This hypothesis is one of the oldest and most influential theories regarding the etiology of depression and has served as a basis for the development of many antidepressants [21,22]. Considering the monoamine theory, it is possible to relate depressive symptoms to genetic changes in neurotransmitters and their transporters [23].

**Figure 1:**
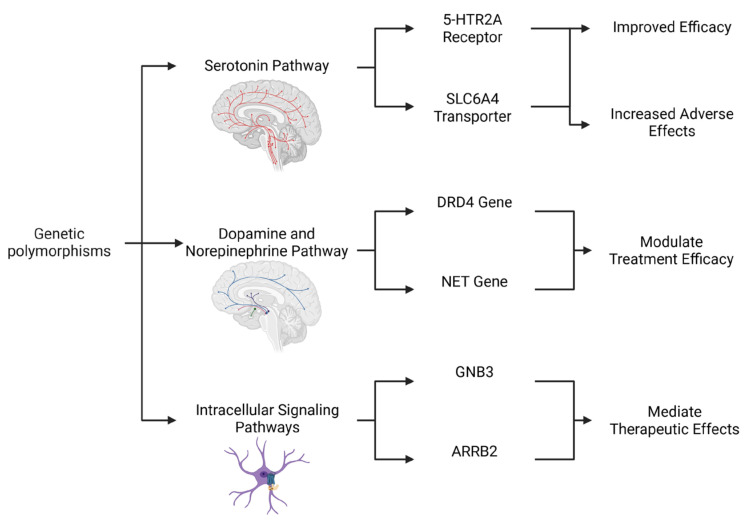
Integrated Genetic Influences on the Monoaminergic System and Antidepressant Response. This diagram illustrates a multifaceted genetic framework for predicting antidepressant outcomes and tolerability. The figure goes beyond single-gene analyses by highlighting the interconnected roles of polymorphisms in three key pathways. The serotonin pathway exhibits variants in the *5-HTR2A* receptor and the *SLC6A4* transporter, with different alleles being able to lead to either improved efficacy or an increased risk of adverse effects. The dopamine and norepinephrine pathway includes genetic variations in the *DRD4* and *NET* genes, which modulate treatment efficacy. Lastly, the Intracellular Signaling Pathways highlight downstream modulators such as *GNB3* and *ARRB2*, which mediate therapeutic effects. Together, these genetic factors contribute to the complex and variable nature of antidepressant response in MDD. Created in Biorender. Bertollo, A.G. et al. (2025).

The presence of genetic polymorphisms can predict the effectiveness of antidepressants (Figure 1; Table 1). A meta-analysis by Lin et al. established that *5-HTR2A* genetic polymorphisms affect antidepressant efficacy in MDD. The analysis showed that variations in the *5-HTR2A* gene, which creates the 5-HT2A receptor, determine patient responses to antidepressants. Moreover, it identified rs6313 T4C and rs7997012 G4A polymorphisms as predicting a positive antidepressant response in MDD but found no such link for rs6311 C4T. The influence of these variants on antidepressant efficacy is not yet fully elucidated. Nevertheless, they may impact the integrity of this system, consequently modulating the serotonin signaling cascades in either a positive or a negative manner. Consequently, this study emphasizes how genetics influences treatment response and validates pharmacogenetics’ role in psychiatric care [22].

**Figure 2:**
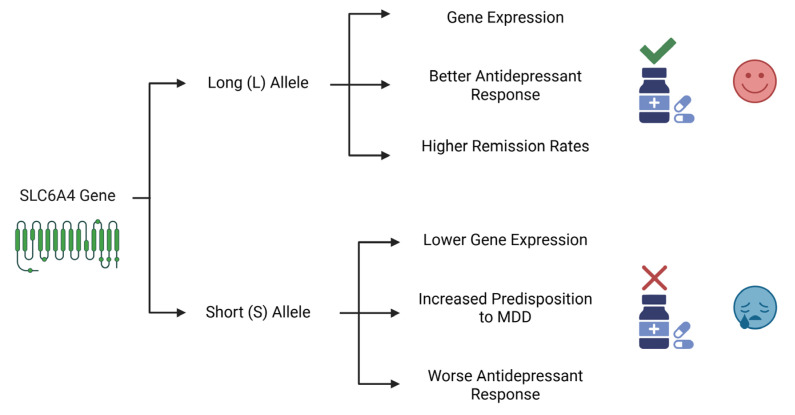
The Differential Impact of *SLC6A4* Gene Variants on MDD Susceptibility and Antidepressant Response. This diagram illustrates the contrasting roles of the two main alleles of the SLC6A4 gene’s promoter region. The long (L) allele is associated with higher gene expression, leading to better antidepressant efficacy and higher remission rates. In contrast, the short (S) allele is linked to lower expression, an increased genetic predisposition to MDD, and a worse therapeutic outcome. Created in Biorender. Bertollo, A.G. et al. (2025).

*SLC6A4* encodes a serotonin transporter (SERT) and regulates serotonergic neurotransmission. A polymorphic variation in the *5-HTT* gene-linked polymorphic region (*5-HTTLPR*) has been identified as being involved in symptoms and poor responses to antidepressants [23]. The long (L) allele, which contains a 44-base-pair insertion and is characterized by a 16-repeat sequence, is associated with higher transcriptional activity of the gene. This leads to a greater expression of the serotonin transporter (SERT) protein compared to that achieved with the short (S) allele. Variants of *5-HTTLPR* are associated with the onset of depression, with the nine-repeat allele being related to cases of major depression with melancholia and cognitive dysfunction [24,25,26,27]. The differential effects of these variants on both genetic predisposition and treatment outcomes are summarized in Figure 2.

The 5-HTTLPR polymorphism influences the remission of depressive symptoms in individuals who developed their first major depressive episode after age 55 and received chronic citalopram treatment. Specifically, the L/L genotype in the *SLC6A4* gene’s promoter region resulted in an 80% remission rate, significantly higher than the 47% rate for other genotypes. Furthermore, LA homozygosity and the LA-12 combination in the *SLC6A4* gene’s promoter predicted the best citalopram responses. Conversely, the S allele of the *SLC6A4* gene is associated with reduced serotonergic function compared to the L allele. Functional studies have demonstrated altered brain responses to serotonergic challenges in older depressed patients who are carriers of the S allele. This is attributed to the influence of *SLC6A4* on SERT expression, which is higher with the L (long) allele and lower with the S (short) allele [26].

A study in the USA highlighted the LA allele as conferring more significant transcription of the *SLC6A4* gene, resulting in increased HTTLPR serotonin transporter levels in the brain and other tissues. This condition may lead to a lower burden of adverse effects for antidepressant medications that target this transporter, such as citalopram [28]. The C/C genotype, homozygous for cytosine in the SNP and serotonin 2A (5-HT2A) receptor (HTR2A) 102 T/C locus, has been associated with side effects during treatment with paroxetine, an SSRI. These side effects led to the discontinuation of paroxetine use. Researchers did not identify these effects with mirtazapine treatments, a non-SSRI antidepressant [29].

A Japanese study compared the clinical responses of paroxetine and fluvoxamine in patients with depression. The results indicated that individuals carrying the *L* allele responded more favorably to SSRIs than those with the *S/S* genotype related to the *5-HTTLPR* polymorphism. Additionally, patients with the *L/L* genotype achieved remission earlier, within two weeks, consistent with findings reported in Western and Chinese studies [30].

The efficacy of escitalopram appears to be influenced by the *5-HTTLPR* polymorphism in an ethnicity-dependent manner. Specifically, a study involving 47 Caucasians participants showed that individuals with the *L/L* genotype experienced more significant reductions in depressive symptoms, as well as higher response and remission rates, compared to a group of 118 Korean participants. This suggests that the *5-HTTLPR* polymorphism may predict escitalopram efficacy differently across ethnicities. Among the white participants, carriers of the *L* allele demonstrated a 50% higher likelihood of responding to and achieving remission after SSRI treatment than the Asian participants in the study [31].

Furthermore, serotonin may be a mechanism underlying sex differences in the prevalence and presentation of disorders related to mood and impulsivity. A study in healthy individuals found that men with tryptophan depletion showed increased impulsivity without impact on mood. Women with tryptophan depletion, on the other hand, adopted a cautious response style associated with depression, presenting a substantial reduction in mood. The sex-linked difference in mood was more pronounced in the *L/L* and *S/S* groups of the *5-HTTLPR* polymorphism. In women, the response to acute tryptophan depletion was more pronounced in the L/L and S/S genotypes. Women with these specific genotypes showed a significant reduction in mood in response to tryptophan depletion. Conversely, in men, acute tryptophan depletion increased impulsivity without affecting mood, regardless of the *5-HTTLPR* genotype. The study suggested that serotonin may be a mechanism underlying sex differences in the prevalence and presentation of disorders related to mood and impulsivity [32]. A study describing sex differences in depression identified a more significant influence of *5-HTTLPR* polymorphisms on the pathophysiology of MDD in women, suggesting a possible sex-specific difference in the genetic modulation of depression by *5-HTTLPR* [33] and its effect on the response to both SSRI and non-SSRI treatments [34].

Therefore, studies have shown mixed results regarding the association between the *5-HTTLPR* polymorphism and the response to antidepressants [28,31], and the complexity of depression suggests that multiple genetic and environmental factors contribute to variability in response to treatment. Therefore, the exclusive use of *5-HTTLPR* as a predictive marker in clinical practice may not be sufficient to provide accurate information about the effectiveness of antidepressant therapy in depressed individuals [35]. Instead, newer research is exploring more comprehensive approaches to predict individual responses to antidepressants better [33,36]. This more holistic understanding of pharmacogenetics could lead to more personalized approaches to treating depression, considering the genetic and biological diversity of patients.

Regarding pharmacogenetics and the response to MDD treatment with SSRIs, a study found that the genetic polymorphism rs1800544 in the *DRD4* gene, which encodes the dopamine D4 receptor, demonstrated a significant link with the response to treatment after six weeks of SSRI use. A combined approach, including neuroendocrine factors, specific clinical characteristics, and genetic polymorphisms, could predict response and remission during SSRI treatment on a large scale, which reached 74.8% and 65.5%, respectively [36].

Studies demonstrate a close link between NE function and severe depression and reveal that the norepinephrine transporter (*NET*) crucially mediates synaptic norepinephrine reuptake. The *NET* gene has 14 exons, covering approximately 45 kb. The genetic variants rs5569 and rs2242446 (*C/C* genotype) are located in exon 9 (1287G/A) of the *NET* gene and represent a silent mutation. One case–control study was conducted to investigate the polymorphic distribution of the rs5569 and rs2242446 variants between the case group (with depression) and the control group, examining the association between these genetic variants and depression in the Chinese Han population. The results revealed significant differences in the genotypic distribution and allele frequency of the rs5569 and rs2242446 loci of the *NET* gene between the case group and the control group, indicating a correlation between these genes and depression. Notably, the *C/C* genotype of these loci was identified as a protective factor against depressive episodes, consistent with previous studies in the Han population of southern China but in contrast to the results of studies conducted in Korea. Therefore, more comprehensive studies with larger samples are needed to confirm the relationship between the *NET* gene and depression [37]. Ryu et al. (2004) reported a significantly lower frequency of the T/T genotype (homozygous for thymine) in the *NET* gene in patients with major depression compared to normal controls when the T-182C polymorphism genotypes were classified into two groups, i.e., a T/T group and a TC + C/C group (homozygous for cytosine) [38]. This also suggests that the T-182C polymorphism in the *NET* gene may be associated with major depressive disorder. However, polymorphisms in the *NET* gene are not associated with suicide risk in individuals with MDD [39].

The *GNB3* gene encodes the *β*3 subunit of the G protein complex, which plays a crucial role in the intracellular signaling cascade following the activation of monoamine receptors, such as the serotonin and norepinephrine receptors. This signaling cascade is fundamental to the therapeutic effects of antidepressants. In pharmacogenetics, genetic variations in the *GNB3* gene, specifically the functional polymorphism C825T (or rs5443), have been associated with the response to antidepressants. This polymorphism’s T/T genotype is significantly associated with a more effective response to the antidepressants escitalopram and nortriptyline, especially with improved neurovegetative symptoms such as sleep and appetite. Furthermore, the *T/T* genotype was related to a lower incidence of treatment-related insomnia and more significant weight gain while using the same medications. These findings highlight the relevance of the C825T polymorphism in the *GNB3* gene as a potential genetic marker to predict the individual response to antidepressants and the occurrence of specific side effects. This more profound understanding of pharmacogenetics may contribute to a more personalized and practical approach to MDD treatment [40].

Still related to the G protein complex, the G-protein coupled receptor *β*-arrestin 2 (ARRB2) gene is involved in the signaling pathways downstream of and associated with the response to antidepressant treatment. Researchers examined five ARRB2 single-nucleotide polymorphisms (SNPs) (rs1045280, rs2036657, rs4790694, rs3786047, and rs452246) in 569 patients (including Caucasian, Afro-Caribbean, and Asian) with a depressive episode treated for six months. The analysis results indicated that patients with the *GG/GT* genotype for rs4522461 and *AA/AC* for rs4790694 showed an inferior antidepressant response compared to other genotypic groups, reinforcing the evidence that *β*-arrestin 2 plays a role in regulating intracellular signal transduction processes involved in antidepressant treatment [41].

**Table 1:** Genetic polymorphisms in neurotransmitters and neurotransmitter transporters and antidepressant response.

Antidepressant	Relevant Genotype	Gene or Enzyme	Metabolism Alteration	Clinical Implications	Ethnicity	Reference
Citalopram	5-HTTLPR L/L	*SLC6A4* (SERT)	Higher remission rate	L/L genotype shows a higher remission rate (80%) compared to other genotypes (47%)	They do not provide data to determine ethnicity	[26]
Paroxetine	C/C at SNP 102 T/C	*5-HT2A*	Elevated side effects	Higher incidence of side effects; no effects found with mirtazapine treatment	They do not provide data to determine ethnicity	[29]
Fluvoxamine	L allele	*SLC6A4* (SERT)	More favorable response	Individuals with the L allele have a better response to SSRIs compared to individuals with the S/S genotype	Chinese Han	[30]
Escitalopram	5-HTTLPR L/L	*SLC6A4* (SERT)	Ethnicity-dependent response	L/L genotype shows a more significant reduction in depressive symptoms in whites compared to Koreans	Chinese Han	[31]
Dopamine D4	rs1800544	*DRD4*	Positive response to SSRIs	Polymorphisms associated with positive response to SSRIs after six weeks of treatment	Caucasians	[36]
NET	rs5569, rs2242446 C/C	*NET*	Protection against depressive episodes	C/C genotype associated with lower risk of depressive episodes; variability found between populations	They do not provide data to determine ethnicity	[37]
GNB3	C825T T/T	*GNB3*	Improved response and side effects	T/T genotype associated with more effective response and less insomnia; increased weight gain	They do not provide data to determine ethnicity	[40]
β-arrestin 2	rs452246 GG/GT	*ARRB2*	Inferior antidepressant response	GG/GT genotypes show inferior antidepressant response compared to other genotypes	They do not provide data to determine ethnicity	[41]

## 5. Pharmacogenetics Related to Neuroplasticity and Growth Factors

Changes in neuroplasticity pathways are intricately linked to the pathophysiology of MDD, significantly influencing antidepressant response [42]. Therefore, it becomes imperative to ascertain the influence of specific genetic factors, particularly those associated with growth factors and neuroplasticity, in determining individual responses to antidepressant therapies (Table 2).

A prospective study [43] identified the role of secondary messenger pathways in antidepressant efficacy, highlighting the previously observed pharmacogenetic contribution of mitogen-activated protein kinase 1 (*MAPK1*) to antidepressant response [44]. Cyclic AMP-responsive element-binding protein 1 (*CREB1*) encodes a transcription factor that is a downstream target of the MAPK pathway, both contributing to neuroplasticity. The study found that in the *MAPK1* gene, the rs6928 SNP positively affected the response to venlafaxine and escitalopram and the remission of depressive symptoms [43].

Brain-derived neurotrophic factor (*BDNF*) is involved in hippocampal neurogenesis, memory [45], neuronal growth [46], and brain plasticity, in addition to negatively impacting recovery, indicating that genetics may play a significant role in emotional vulnerability and the recovery process from stressful events in older ages [47]. MDD reduces its levels, despite its wide distribution in the central nervous system [42,46]. A case–control study found that six SNPs in the *BDNF* gene are associated with the diagnosis of MDD, namely, rs12273539, rs11030103, rs6265, rs28722151, rs41282918, and rs11030101. The rs61888800 polymorphism contributes to better antidepressant effects with desipramine and fluoxetine [19].

**Table 2:** Genetic polymorphisms in neuroplasticity and growth factors and antidepressant response.

Gene or Enzyme	Relevant Genotype	Pathway or Function	Effect on Antidepressant Response	Clinical Implications	Ethnicity	References
MAPK1	rs6928	MAPK pathway, neuroplasticity	Positive effect on response and remission with venlafaxine and escitalopram	Enhances the effectiveness of antidepressants by influencing neuroplasticity	They do not provide data to determine ethnicity	[43]
*BDNF*	rs12273539, rs11030103, rs6265, rs28722151, rs41282918, rs11030101	Neurogenesis, brain plasticity	Associated with MDD diagnosis; rs61888800 contributes to better antidepressant effects with desipramine and fluoxetine	Variations may impact emotional vulnerability and recovery; treatment effects are genotype-dependent	Mixed-race (Mexican American) and Hispanic	[19,45,46]
TrkB	rs2289657, rs56142442	*BDNF* receptor, neuroplasticity	Positive response to desipramine treatment in depressed patients	Genetic variations may enhance antidepressant efficacy by influencing neuroplasticity	Mixed-race (Mexican American)	[19]

The *BDNF* receptor tropomyosin receptor kinase B (TrkB) is also essential in the pathophysiology of MDD and the therapeutic mechanisms of antidepressants [48]. The genetic variations rs2289657 and rs56142442 (homozygosity for the *C* allele in SNPs, synonymous with the *TrkB* gene) are associated with a more positive response to desipramine treatment in depressed patients [19].

A study identified a specific locus on chromosome 17 around the *ETV4* gene associated with antidepressant responses. Previous research, using neuronal cell lines and mouse models, linked *ETV4* to *BDNF*-induced hippocampal dendritic development and plasticity. The implication of *ETV4* in the response to antidepressants is in line with the hypothesis that antidepressants exert their effects by promoting hippocampal neuroplasticity [49].

Vascular endothelial growth factor (VEGFA), a key player in angiogenesis and neuroprotection, has also been implicated in the response to antidepressant treatment. Our study has revealed that one of the antidepressant mechanisms of ketamine is related to the increase in VEGFA [50]. However, treatment with SSRIs does not modulate the VEGFA levels [51], indicating the need for more studies relating to the effect of antidepressants on VEGFA. These findings could potentially pave the way for the development of more targeted and effective antidepressant therapies.

## 6. Pharmacogenetics Related to the Inflammatory Response

Inflammation, specifically neuroinflammation, contributes to the pathophysiology of MDD [52,53,54]. Polymorphisms in genes associated with the inflammatory response may influence antidepressant effectiveness, as several inflammatory markers are chronically altered in individuals with the disorder [2,55,56,57] (Table 3).

The tumor necrosis factor-alpha (TNF-α) gene is related to the function of the serotonin transporter, the inhibition of neurogenesis in the hippocampus, and the inflammatory response. Therefore, it has implications for both the pathophysiology and the treatment of MDD [58,59]. Furthermore, other pro-inflammatory cytokines and immune response pathways, such as IL-6, IL-11, and the HPA axis, contribute to the condition and influence the response to antidepressants [60]. One study identified that increased levels of TNF-α are associated with a lack of response to escitalopram. This association extends to TNF-related genes, such as lymphotoxin alpha (LTA), formerly TNF-*β*, which also act as biomarkers for the response to escitalopram. The study also found that, about the gene that transcribes IL-11, carriers of the A allele (genetic variant rs1126757) are more likely to respond adequately to escitalopram, also showing a 50% reduction in IL-11 expression compared to homozygous carriers of the *G* allele [56].

Regarding the use of duloxetine in cases of MDD, IL-6 gene variants, specifically rs2066992 and rs10242595, were nominally associated with the response to duloxetine treatment. Carriers of the T allele of rs2066992 responded better to the treatment, indicating a possible functional consequence of the variant. The high linkage disequilibrium (LD) between rs2066992 (located in the intronic region of IL-6) and a 5′UTR regulatory variant (rs1800796) suggests a possible role for rs2066992 in regulating gene expression. The results indicate that genetic variants in IL-6 may play a role in predicting the response to duloxetine and a placebo, suggesting a common underlying mechanism for drug targets and the endogenous response [2].

Another relevant area of research explores the interaction between genetic variations in the HPA system and the effectiveness of antidepressant treatment. A study identified an SNP in the corticotropin-releasing hormone receptor 1 (*CRHR1*) gene associated with antidepressant remission during SSRI and SNRI treatment in a Chinese Han population. The results showed that the rs28364032 variant of CRHR1 contributes to antidepressant remission. Furthermore, functional assays demonstrated that the change from G to A in rs28364032 reduces the protein expression of CRHR1, which may be one of the mechanisms by which it contributes to an excellent response to treatment [55].

Thirty-five genes associated with remission in response to antidepressants were identified, using an analysis of rare variants in whole-exome genotyping data, and some identified genes have implications in inflammatory and neuropsychiatric processes, such as methyltransferase like 3 (*METTL3*), 26S subunit proteasome, non-ATPase 13 (*PSMD13*), tyrosine kinase 2 (*TYK2*), alpha-1,3-glucosyltransferase (*ALG6*), spliceosome-associated protein homolog (*CWC22*), family pyrin domain-containing 1 (*NLRP1*), and olfactory receptors. These genes play diverse roles, such as in DNA methylation, the response to depression treatment, IL-6 signaling, mutations associated with neurological disorders, the assembly of the exon junction complex, and mood regulation through the olfactory system, highlighting the interaction of inflammatory factors in response to antidepressants [61].

A study of 755 individuals on SSRIs and SNRIs indicated that genetic factors can influence the response to antidepressants independently of the systemic inflammation levels. This effect was especially pronounced when a significant interaction was observed between the antidepressant and the polygenic risk score for CRP (CRP-PRS). This metric reflects a genetic predisposition to systemic inflammation. Despite this, it is suggested that when genetic factors are permanently affected by systemic inflammation, they may induce compensatory processes that act in opposition. However, the exact mechanism of these processes is not yet fully elucidated [57].

**Table 3:** Genetic polymorphisms in inflammatory response and antidepressant response.

Gene or Enzyme	Relevant Genotype	Pathway or Function	Effect on Antidepressant Response	Clinical Implications	Ethnicity	References
TNF-α	Not specified	Inflammatory response, serotonin transport	Increased TNF-α levels are associated with a lack of response to escitalopram; LTA is also a biomarker for escitalopram response	Variants may influence response to SSRIs; TNF-α impacts antidepressant effectiveness	They do not provide data to determine ethnicity	[56,60]
IL-6	rs2066992, rs10242595	Inflammatory response, gene regulation	Carriers of the T allele of rs2066992 show better response to duloxetine	Variants in IL-6 may help predict response to duloxetine	Caucasians	[2]
CRHR1	rs28364032	HPA axis, stress response	Associated with antidepressant remission; the A allele reduces CRHR1 protein expression	Genetic variation may enhance antidepressant response by influencing stress response pathways	They do not provide data to determine ethnicity	[55]
Various genes (e.g., *METTL3*, *PSMD13*, *TYK2*)	Not specified	Inflammatory and neuropsychiatric processes	Identified in rare-variant analysis related to antidepressant remission	Suggests the interaction of inflammatory factors in antidepressant response	Caucasians	[61]

## 7. Pharmacogenetics Related to CYP450 and Antidepressant Metabolism

**Figure 3:**
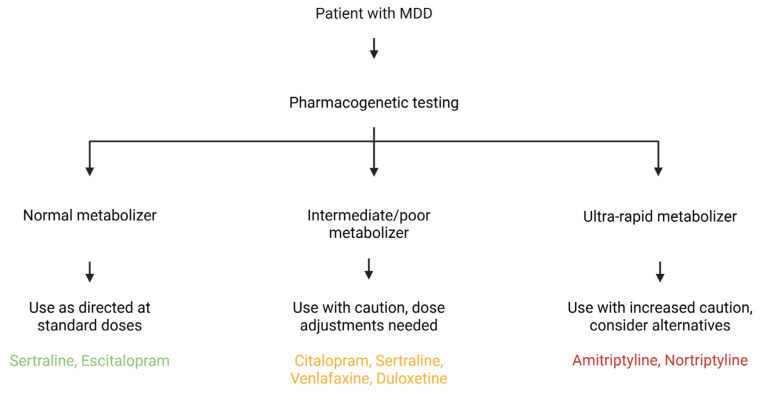
A Multifaceted Framework for Pharmacogenetic-Guided Antidepressant Treatment. This diagram integrates clinical pharmacogenetic recommendations with the broader biological mechanisms that influence antidepressant response. The central flowchart illustrates how a patient’s metabolism phenotype for *CYP450* enzymes, such as *CYP2D6* and *CYP2C19*, directly informs a clinical course of action. This is further contextualized by interconnected panels that highlight the roles of genetic variations in neurotransmitter systems (e.g., *SLC6A4*, *DRD4*) and pathways related to neuroplasticity and inflammation (e.g., *BDNF*, IL-6). The figure highlights that an optimal therapeutic strategy necessitates a comprehensive understanding of a patient’s genetic profile to enhance efficacy and minimize the adverse effects. Created in Biorender. Bertollo, A. G. et al. (2025).

*CYP450* is a family of liver enzymes responsible for metabolizing various substances, including many antidepressants. Genetic variations in the genes that encode these enzymes [62] significantly impact the serum concentrations of antidepressants and, consequently, can modulate the actions of these drugs, either increasing or decreasing their effect [63] (Table 4). The study of the *CYP450* genotype contributes to a more appropriate and individualized drug choice, minimizing the rates of treatment resistance and adverse effects [64]. Furthermore, pharmacogenetics contributes to influential drug associations in MDD cases resistant to monotherapy treatment [65]. The potential clinical utility of this information is significant, as it can inform personalized treatment decisions and improve patient outcomes. A proposed workflow for incorporating pharmacogenetic testing into clinical practice is illustrated in Figure 3.

Antidepressants from different classes may have their effects influenced by genetic variations in the *CYP450* family. A significant portion of the variation in the release and elimination of nortriptyline, a TCA, can be directly attributed to the number of functional *CYP2D6* genes, as well as to the *CYP3A4*, *CYP2C19*, and *CYP1A2* enzymes [66]. In the case of mirtazapine, individuals with the *CYP2B6* *6/*6 genotype have probably reduced enzyme function, reducing mirtazapine metabolism, and therefore have a more pronounced reduction in depressive behavior [67].

One study analyzed the association of poor (individuals who have homozygous alleles with loss of function and consequent absence of the enzyme) and intermediate (individuals carrying genotypes associated with substantially reduced, but not completely abolished, enzymatic capacity) metabolizer statuses linked to *CYP2C19* and *CYP2D6* with exposure to antidepressants and identified that these variations interact uniquely with each medication. Subjects with the *CYP2D6* poor metabolizer or intermediate metabolizer phenotypes exhibited a 48% increase in aripiprazole exposure compared to normal metabolizers. Regarding escitalopram oxalate, poor metabolizers had a 163% increase in drug exposure compared to subjects who were normal metabolizers, which appeared to be related explicitly to *CYP2C19* metabolism. Finally, intermediately metabolizing individuals exhibited a 38% increase in sertraline hydrochloride exposure compared to normal metabolizers (NMs), which was associated with *CYP2C19* metabolism [68].

Another study identified the relevance of *CYP2D6* and *CYP2C19* pharmacogenetics in personalizing the use of antidepressants, such as nortriptyline, citalopram, amitriptyline, sertraline, and paroxetine, focusing on how genotypes influence efficacy and adverse drug reactions (ADRs). Among 52 cases, only 15% had NM phenotypes for *CYP2D6* and *CYP2C19*, indicating that 85% of the patients had non-NM phenotypes, predisposing them to either ADRs or ineffective responses. In 45 cases analyzed for the *CYP2D6*/*CYP2C19*-antidepressant response, 57% were *CYP2D6* non-NMs, and 62% were *CYP2C19* non-NMs, which aligns with earlier findings. Researchers deemed 48% of the *CYP2D6*/*CYP2C19*-antidepressant response–ADR pairs and 21% of the *CYP2D6*/*CYP2C19*-antidepressant response–ineffectiveness pairs actionable, suggesting that pharmacogenetic testing could mitigate half of the ADRs and one-fifth of the ineffective responses. In other words, these findings emphasize that while the impact of pharmacogenetics on antidepressant efficacy (~20%) is modest, its influence on tolerability (~50%) is significant, underscoring the value of *CYP2D6* and *CYP2C19* genotype screening to enhance treatment safety and the efficacy of antidepressants [69].

**Table 4:** Genetic polymorphisms related to CYP450 and antidepressant response.

Gene or Enzyme	Function or Pathway	Impact on Response	Clinical Implications	Ethnicity	References
*CYP2D6* (Poor metabolizer)	Metabolism of various antidepressants	Variable drug effect: increased/decreased	Risk of treatment resistance and adverse effects; pharmacogenetic testing recommended	Caucasians	[62,63,65]
*CYP2C19* (Poor metabolizer)	Metabolism of drugs like escitalopram	Increased drug exposure	Elevated risk of adverse effects; personalized dosing necessary	African ancestry	[68]
*CYP3A4* and *CYP1A2*	Metabolism of antidepressants like nortriptyline	Affects drug release and elimination	Requires dosing adjustments based on genotype	Caucasians	[66]
*CYP2B6* (6/6 genotype)	Metabolism of mirtazapine	Reduced enzyme function; increased effect	Greater reduction in depressive symptoms; dosage adjustments may be needed	They do not provide data to determine ethnicity	[67]
*CYP2B6* (6/6 genotype)	Metabolism of mirtazapine	Reduced enzyme function; increased effect	More significant reduction in depressive symptoms; dosage adjustments may be needed	They do not provide data to determine ethnicity	[67]

Genetic variations within the cytochrome P450 (*CYP450*) family are commonly used as an exclusion criterion in clinical research. This is mainly because slow metabolizer variants of the *CYP2D6* gene, such as alleles 3(A), 4(B), and 5(D), are linked to an increased risk of adverse events due to their decreased or absent metabolic activity [70,71]. Given the significant percentage of individuals with *CYP450* genetic variants in Sub-Saharan Africa, a region with a high degree of gene diversity, clinical studies have historically lacked representation from these populations. This poor inclusion hinders the development of more effective medications for these communities [72]. It underscores the critical need for comprehensive research that includes extensive and ethnically diverse samples to ensure an appropriate outcome for different groups [70]. The distribution and frequency of these genetic variations differ significantly across ethnic and geographic populations. For instance, the poor metabolizer phenotype for *CYP2D6* is present in 5–10% of the white population but is rarer in the black and Asian populations. In contrast, the poor metabolizer phenotype for *CYP2C19* is significantly more prevalent in Asian populations (about 20%) compared to European populations (2–5%) [12,71].

## 8. Final Considerations and Future Directions

Genes and genetic polymorphisms are inherently linked, regulating gene expression and determining organismal characteristics. This intricate interplay is particularly crucial in understanding the genetic predisposition to depression, the variability in the response to antidepressant treatment, and the identification of potential therapeutic targets.

The genetic influence on antidepressant response is complex and may vary according to the specific genes, ethnicity, and neurotransmitters considered. It is essential to consider that research with small samples does not usually demonstrate significant differences between carriers of genetic alterations and individuals without these alterations [35]. Still, these differences are evident in research with larger samples [36]. Researchers have demonstrated in different samples that ethnicity influences the effect of polymorphisms on drug response [33], and thus, we must consider ethnic differences. Moreover, sex also influences this, with genetic polymorphisms associated with MDD predominating in women [33,72]. Although several researchers have studied genetic polymorphisms in the *SLC6A4* gene, their impact on the pathophysiology of MDD has yet to be elucidated.

It is necessary to improve the number of studies on other neurotransmitters, such as dopamine and noradrenaline, and their transporters. Still, research has failed to replicate previously verified results, possibly due to differences in the antidepressant analyzed or its class [73]. Therefore, more research with large samples is needed to fully understand how individual genetic variations can affect the effectiveness of antidepressant treatments and how this can inform clinical practice in the area of mental health.

The intersection of pharmacogenetics, neuroplasticity, and growth factor research has yielded valuable insights for personalized approaches to depression treatment. Understanding genetic variations linked to neuroplasticity-related genes, such as *BDNF* and VEGFA, holds the promise of tailoring the most effective antidepressant interventions for individuals with MDD. Similarly, alterations in the inflammatory response contribute to the disorder and may be related to the pharmacogenetics of the reaction to classical antidepressants.

The *CYP450* family of hepatic enzymes plays a crucial role in the metabolism of several antidepressants, with genetic variations in these genes responsible for significant impacts on the serum concentrations of these drugs. In turn, the serum concentrations can modulate the effects of antidepressants, increasing or decreasing their effectiveness. The study of the *CYP450* genotype contributes to a more appropriate and personalized choice of medications, minimizing the rates of treatment resistance and adverse effects. The influence of the genetic variations in the *CYP450* family extends to different classes of antidepressants, affecting the release and elimination of substances. However, an exclusion criterion in clinical investigations about genetic variations presents challenges, especially in regions such as Sub-Saharan Africa, where the lack of comprehensive studies makes the access to more effective medicines difficult, highlighting the need for extensive research to ensure appropriate results for different ethnicities.

In the context of clinical application, the recommended pharmacogenetic tests for antidepressants focus on two key biological systems [74]. The best-established and most clinically relevant tests examine genes that code for drug-metabolizing enzymes, primarily within the *CYP450* system. These tests, which include genotyping for *CYP2D6* and *CYP2C19* variants, help predict how quickly a patient will metabolize a drug, guiding dosage to minimize the adverse effects and ensure therapeutic levels. Genetic variants in these genes can classify patients into different metabolic profiles, such as poor, intermediate, standard, rapid, or ultra-rapid metabolizers, which directly affect drug efficacy and the risk of side effects. Following genotype-informed recommendations, particularly for drugs like escitalopram, sertraline, and paroxetine, can reduce adverse drug reactions and improve treatment efficacy, especially in patients who have experienced prior treatment failure [75,76].

Additionally, significant progress has been made in testing drug targets, which are the genes related to the pharmacological action of the antidepressants themselves. These include pharmacodynamic genes such as *SLC6A4* and *BDNF*. For *SLC6A4*, genetic variants like the *5-HTTLPR* polymorphism have been associated with antidepressant response, with the L allele predicting better response and remission, especially in Caucasian populations. In the *BDNF* gene, the Val66Met (rs6265) polymorphism is also associated with a better SSRI response, particularly in Asian populations [77]. Beyond these variants, epigenetic modifications such as DNA methylation of the SLC6A4 and *BDNF* genes show promise as biomarkers for predicting treatment outcomes [78]. However, despite these promising findings, the evidence is often inconsistent and not yet robust enough for routine clinical decision-making. While integrating information from both metabolic genes and these pharmacodynamic markers could theoretically enhance personalized treatment strategies in the future, the current clinical guidelines do not yet support the routine use of *SLC6A4* or *BDNF* testing for the prescription of antidepressants [79].

Studies suggest that pharmacogenetic analyses help avoid adverse effects that specific gene variations may cause or exacerbate [80,81,82,83]. A pragmatic randomized clinical trial evaluated the effectiveness of a particular, commercially available pharmacogenetic test to guide antidepressant prescribing at 22 sites in the United States. The results indicate that healthcare professionals require more excellent education and support to implement pharmacogenetics in clinical practice, particularly for antidepressant prescription, and the study’s conclusion reveals the practice’s promise [65].

Studies have shown that polymorphic variants of SERT and *BDNF* individually contribute to severe and resistant depression in people who suffer from childhood stress. Further, researchers discovered that variations in both SERT and *BDNF* genes modify the risk of depression conferred by childhood maltreatment when analyzed together [84]. Future studies should investigate the interaction between consistent polymorphisms and environmental factors, offering an interesting direction for research in this area.

Pharmacogenetics related to antidepressants seems to offer a promising possibility of personalizing antidepressant treatments based on the individual genetic makeup of patients. This can be particularly beneficial due to the high rate of non-responsiveness to classic treatment strategies, improving the effectiveness of antidepressants and providing a more efficient path to recovery for patients with depression. However, although some results have highlighted the importance of pharmacogenetics for treatments, others point to the need for further investigations, observing the interaction between genetic variables and other biological or environmental variables.

## Data Availability

No new data were created or analyzed in this study. Data sharing is not applicable to this article.
